# Female resistance and harmonic convergence influence male mating success in *Aedes aegypti*

**DOI:** 10.1038/s41598-019-38599-3

**Published:** 2019-02-14

**Authors:** Andrew Aldersley, Lauren J. Cator

**Affiliations:** Department of Life Sciences, Imperial College London, Silwood Park, Buckhurst Road, Ascot, SL5 7PY UK

## Abstract

Despite the importance of mosquito mating biology to reproductive control strategies, a mechanistic understanding of individual mating interactions is currently lacking. Using synchronised high-speed video and audio recordings, we quantified behavioural and acoustic features of mating attempts between tethered female and free-flying male *Aedes aegypti*. In most couplings, males were actively displaced by female kicks in the early phases of the interaction, while flight cessation prior to adoption of the pre-copulatory mating pose also inhibited copulation. Successful males were kicked at a reduced rate and sustained paired contact-flight for longer than those that were rejected. We identified two distinct phases of acoustic interaction. Rapid frequency modulation of flight tones was observed in all interactions up to acceptance of the male. Harmonic convergence (wingbeat frequency matching) was detected more often in successful attempts, coinciding with the transition to stabilised paired flight and subsequent genital contact. Our findings provide a clearer understanding of the relationship between acoustic interactions and mating performance in mosquitoes, offering insights which may be used to target improvements in laboratory reared lines.

## Introduction

Mass release of sterile and transgenic males to manage wild mosquito populations is currently being integrated into vector control programmes^1–4^. Ensuring that released males are able to effectively compete for and mate with wild females is a key component of these initiatives^5^. Releasing males with high mating success will facilitate programme success and ultimately improve the feasibility and sustainability of these control strategies in the long-term.

Numerous species of mosquito are known to mate in aerial swarms^6–9^. In the yellow fever mosquito, *Aedes aegypti*, small groups of males aggregate in close proximity to vertebrate hosts^10–13^. Although the precise mechanisms that mediate swarm formation in this species are not fully understood^9,14^, both chemical and acoustic cues have been proposed^15–17^. Individual females approach—likely in search of a blood-meal source^10,13^—and enter swarms in much lower numbers^11^, leading to a sex ratio that is skewed towards males^8,9^. Males detect a nearby female via the sounds produced by her beating wings^18–20^, and pursue and seize her in mid-air. At this point a quick reorientation takes place such that the pair are “venter-to-venter”^12,21^, a position that facilitates genital engagement. Copulation—which can take between 9 and 31 seconds to complete^12^—has been observed both inside and outside the swarm, and with the pair in flight or at rest^10,22,23^. Since female *Ae. aegypti* predominantly mate only once in a lifetime^24,25^, it is thought that the likelihood that any particular male will successfully copulate is low compared to the total number participating within the group. Mosquito swarms have thus been said to resemble lek mating systems^15,26^, with males clustering around and competing within locations attractive to receptive females^27^, who approach and are mated, while receiving no paternal care contribution^15^.

Fundamentally, successful mating inside a swarm requires males to detect and intercept females in flight (coupling), engage their genitalia (copula formation), and transfer semen (insemination)^12^. Insemination rate is typically used to assess male performance^28–39^, yet the specific behavioural differences that underlie variation in success are poorly defined. In particular, the role of female choice and the degree to which it influences mating outcome is not known. Indeed, the absence of readily observable selective behaviours within mating swarms has raised the question of whether females exert any choice whatsoever in this system^6,22,26^. There are, however, multiple published accounts of females displaying a variety of active behaviours to evade, deter, or displace prospective partners including evasive flight manoeuvres, leg pushes, kicks, or thrusts, and abdominal jerks or tilts^18,21,40–45^. In fact, when individual mating attempts are observed, the proportion that lead to successful copula formation is consistently relatively low (16%^40^, 28%^43^, and 37%^42^).

Until recently, the mechanisms that mediate this apparent choice have remained somewhat elusive. One possible insight into this process has emerged through the study of acoustic signalling between pairs during the mating interaction. Male mosquitoes possess an acute sense of hearing^46,47^ and are strongly attracted towards the sound of the female wingbeat^18–20,48,49^, which they use to locate mates within swarms^6,12^. Phonotaxis towards female-like tones is associated with rapid frequency modulation (RFM) of the male wingbeat^48,49^. When flying males and females are in close proximity to one another they engage in a dynamic sound interaction during which each individual actively modulates their wingbeat frequency in response to the other^50^. In some pairs, this results in an overlap between male and female frequencies at a harmonic ratio^51–54^. This interaction, termed “harmonic convergence”, is prevalent among opposite-sex couples of a range of species^43,50–53^. In *Ae. aegypti*, it is significantly correlated with mating success^40^. Thus, acoustic signalling—and specifically harmonic convergence—has been proposed as a mechanism by which females may assess and ultimately determine whether to mate with a particular male. It is unclear if and how harmonic convergence and RFM relate to one another^49^, or how they integrate into the behavioural mating sequence.

In order to build a complete picture of what drives differential mating success in mosquitoes, a more detailed knowledge of the entire mating process—including the pre-copulatory phases that lead to the formation of a mating pair and the effect of female choice activities—is needed. This information is currently lacking, at least in part, due to the limitations of observing these rapid mid-air mating interactions. In particular, the steps required for a male to form a copula with the female have been largely overlooked as an important component of the mating sequence, despite being recognised as vital for achieving genital contact^21^.

Here, we investigated the role of acoustics in mating interactions in *Ae. aegypti*. We developed a system to simultaneously record high-speed video and sound from interactions between free-flying males and tethered-females. Through analysis of the structure of behavioural and acoustic interactions during mating attempts, we explored how different aspects of this process relate to the likelihood of female rejection and ultimately, male mating success (or failure). Most attempts were unsuccessful. Males were actively rejected by female kicks in the early post-contact stages of an interaction. Those that eventually succeeded in mating were kicked at a lower rate and remained in paired flight for longer than those that were rejected. Flight cessation prior to the adoption of the venter-to-venter position significantly decreased mating success. Moreover, harmonic convergence events were more likely to occur—and in greater number—in successful mating interactions, between coupling and genital contact and specifically as the pair transitioned into the final mating pose. These results highlight the importance of female rejection behaviours and support the importance of acoustic interactions for driving mating outcomes in *Ae. aegypti* mosquitoes.

## Results

**Figure 1:**
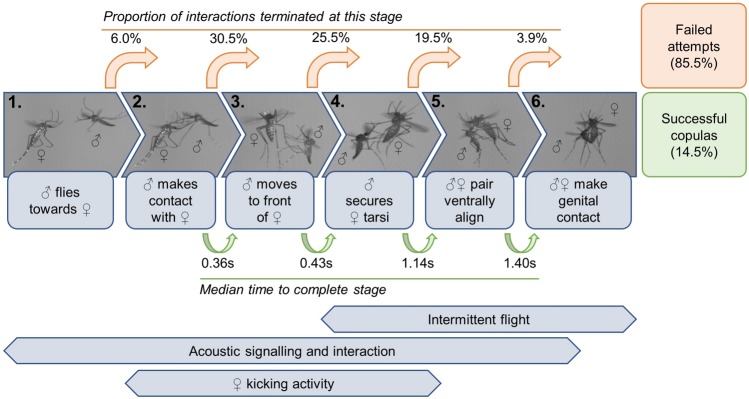
Flow diagram of the steps observed in mating interactions between free-flying male and tethered female *Ae. aegypti* mosquitoes (further detailed in Table 1). To make genital contact the pair must sequentially pass through each stage. Rejections (unsuccessful mating attempts)—which can be “active” or “passive” (Table 1)—may occur at any point post-contact and pre-genital engagement. Proportions above each “termination” arrow indicate the percentage of total mating attempts that failed at this stage (a full breakdown for each rejection type is given in Table 3). Green arrows give the median time taken to complete each stage, across all interactions that did so. Acoustic signalling, female kicking activity, and individual flight cessation feature across the span of the interaction.

We recorded a total of 282 interactions from 117 unique pairs of free-flying males and tethered females. A single interaction was defined as any period of continuous contact during which the pair did not physically separate for more than 0.5 s (if, for example, contact was momentarily broken as the pair manoeuvred into position). Multiple interactions occurred in 70.1% of recorded pairs. Interactions consisted of a set of behaviours occurring during specific stages, which were classified according to outcome (Fig. 1, Table 1).

**Table 1:** Observations made (ordered approximately chronologically) and measurements derived from high-speed video recordings of semi-tethered mosquito interactions.

Behaviour		Definition and measurement
Approach and contact direction		The approach and contact direction of the male relative to the tethered female’s body position (frontal, rear, or lateral).
Male hovering		A binary measure indicating whether the male hovered in front of the female before intercepting her (Video S1).
Male interception point		The time at which the male made physical contact with the female, typically initiated by a characteristic tarsal grab (Video S1). Used as a reference point for the beginning of the interaction proper, from which other events are defined.
Female kicking		Any kicking, flicking, or thrusting performed by the female with the use of her legs (Video S3). The timing of each kick event was recorded.
Kick period		Time between first and last kick
Kick rate		The number of kicks/kick period
Male frontally positioned		The time at which the male oriented in the dorsal plane of the female (Video S2).
Male secured tarsal hold		The time at which male obtained sustained and controlled tarsal contact with female (Video S2).
Male ventrally aligned		The time at which the pair adopted the “venter-to-venter” position^11,18,21^, whereby the male achieves an elevated position beneath the female, holding both her mid- and hind-tarsi with his fore- and mid-legs (Video S2, Video S4).
Flight intervals		The start and end time of any interval of flight intermittency by the male or female whilst in contact with one another.
Interaction end		The time at which contact was broken between male and female.
Outcome	Copula (*n* = 41)	An interaction in which genital contact between male and female was achieved (regardless of successful insemination, Video S4).
	Active rejection (*n* = 142)	An interaction that did not result in genital contact and which was terminated by female kicking activity leading to displacement of the male (Video S3). Males in these interactions always received at least one kick.
	Passive rejection (*n* = 77)	An interaction that did not result in genital contact and which was terminated by the male breaking contact with the female, usually after a period of sustained female non-flight (Video S5). These interactions may proceed through all pre-copulatory behavioural stages, but were terminated with the male “grossly misaligned”^21^.
	Male accepted (*n* = 5)	An interaction that was terminated at the ventral alignment stage, but that did not result in genital contact.
	No contact (*n* = 17)	An interaction in which the male approached but made insufficient contact with the female to initiate further positioning.

Once released into the arena males flew freely performing tight loops in the stereotypical “patrolling” pattern of swarming^12^, immediately accelerating towards flying tethered females when they passed within ~10 cm of her position. In most cases (89.4%) females were approached from the frontal or dorsal planes (Table 2). To initiate contact, all males performed a characteristic “grabbing” motion with their legs once they were within ~1–2 body lengths of the female (Fig. 1, Video S1). Most contacts (72.7%) were made from the same direction as the initial approach (Table 2, Fisher’s exact test; *p* = 5.87 × 10^−39^). Males repeatedly attempted to grab the female until adequate tarsal contact had been achieved.

**Table 2:** Count data of approach and contact direction for each recorded interaction.

		Contact direction		
		Frontal	Lateral	Dorsal
Approach direction	Frontal	101	13	5
	Lateral	5	13	12
	Dorsal	16	26	91

A significant association was found between the direction that a male approached and contacted a female from (Fisher’s exact test, *p* = 5.87 × 10^−39^).

Having seized a tethered female, the male began manoeuvring into a ventral position^12^ (Video S2). From first contact, males crawled to the female’s front using her tarsi for leverage (median time 0.36 s, IQR 0.17–0.68 s), then securely grasped her mid- and hind-legs (median time 1.07 s, IQR 0.57–1.64 s), before adopting the venter-to-venter—or ventrally aligned—position (median time 2.26 s, IQR 1.36 s–5.28 s) and making genital contact (median time 4.58 s, IQR 2.94–12.46 s), the point of copula formation. The median time spent in copula was 11.75 s (IQR 9.66–14.48 s). Not all steps were completed in all interactions; failure of the attempt via female rejection of the male occurred at all stages between interception and copulation (Fig. 1, Table 3). Overall, only 14.5% of interactions led to copula formation (Table 1). Termination of genital engagement was instigated by the female through forcible detachment of the male with thrusts of her legs. Dissection of female spermathecae (in the second experimental replicate) confirmed that 100% of interactions scored as copulas (*n* = 20) led to successful insemination. After copulation, males resumed flying in the characteristic swarm style. Consistent with previous findings, males did not approach stationary females^18,55^, even those that had been previously visited, and even if they passed within millimetres of her body.

**Table 3:** Comparison of various measured interaction features for different outcome types, also showing result of Kruskal-Wallis rank tests to check for differences in the given variable (*χ*^2^ statistic and *p*-value).

Measured feature		Interaction outcome						χ^2^	*p*-value
		Copula		Passive rejection		Active rejection			
Total number of outcomes observed		41		77		142		—	—
Median (IQR) contact time (s)		21.45 (16.41, 26.24)		5.33 (2.57, 11.55)		0.84 (0.33, 1.56)		131.63	**2.61 × 10** ^**−29**^
Mean (±sd) number kicks		5.63 ± 4.96		5.43 ± 4.60		5.06 ± 4.32		0.55	7.61 × 10^−1^
Median time to first kick (s)		0.17		0.13		0.11		7.49	**2.37 × 10** ^**−2**^
Median duration of kick period (s)		1.14		0.83		0.68		6.35	**4.17 × 10** ^**−2**^
Median kick rate (kicks/s)		4.74		6.86		7.41		8.35	**1.54 × 10** ^**−2**^
Number exhibiting flight cessation		39		74		14		—	—
Median (IQR) time to cessation (s)		2.74 (1.79, 5.39)		1.47 (1.05, 2.23)		1.05 (0.56, 2.03)		24.92	**3.87 × 10** ^**−6**^
Median time (s) |% terminated	Move to front	0.30	—	0.49	10.3%	0.34	54.9%	3.93	1.40 × 10^−1^
	Secure tarsal hold	1.12	—	0.97	18.2%	1.17	39.4%	—	—
	Ventrally align	2.28	—	3.19	64.9%	2.26	3.5%	—	—
	Genital contact	4.58	—	—	6.5%	—	2.1%	—	—

In each case, normality of measures was tested using Shapiro-Wilk tests. When calculating contact times, only those interactions that concluded before the end of the video recording period (*n* = 234) were included. The median kick period and kick rate tests include only those interactions that involved more than one kick (i.e. kick period ≥ 0, *n* = 215). Flight cessation is classified as the point at which paired flight ended (i.e. when either individual stopped). For rejections, the median time to each pre-copulatory behaviour identified in Fig. 1 is given for those interactions in which the stage was completed. Also presented is the proportion of all rejections that occurred between each step. Kruskal-Wallis tests were not performed on time measurements for males securing female tarsi and paired ventral alignment due to the small sample sizes involved.

### Female rejection rates are high and associated with mating outcomes

**Figure 2:**
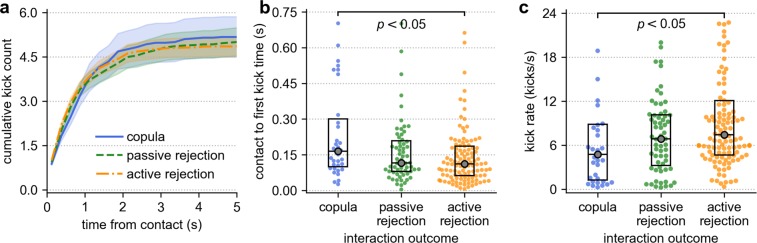
Kicking activity in different interaction outcome types. (**a**) Mean (±standard error) cumulative kick count plotted as a function of time from male-female contact. Most kicks take place within the first seconds of interaction, and at a similar rate across outcome types. (**b**) The distribution times at which the female first kicked the male, relative to contact, for each outcome type. (**c**) The kick rate (number of kicks divided by the kick period, Table 1) for different outcomes, only including interactions that involved more than one kick (kick period > 0). Boxes represent the median and interquartile range for each measure. In comparison to attempts that resulted in active rejection of the male, kicks began significantly later in copulas (Table 3, Dunn’s post-hoc test with Bonferonni correction; *χ*^2^ = 2.64, *p* = 1.26 × 10^−2^) and were delivered at a lower rate (Table 3, Dunn’s post-hoc test with Bonferroni correction; *χ*^2^ = −2.72, *p* = 9.68 × 10^−3^).

The manoeuvring phase immediately succeeding contact was characterised by the female performing rejection kicks at a high frequency (Fig. 2a, Video S3). Kicking occurred in interactions of all outcome categories. Attempts in which they led to displacement of the male were classified as active rejections (Table 1). The number of kicks delivered was strongly associated with the interaction result (*χ*^2^ test for independence with Monte-Carlo resampling; *χ*^2^ = 66.03, *p* = 6.42 × 10^−3^) and, despite the similarity in average kick count across groups (Fig. 2a, Table 3), its distribution varied considerably with outcome (Fig. S2). Moreover, the onset of kicking was delayed in successful mating attempts (Fig. 2b, Table 3, Dunn’s post-hoc test with Bonferroni correction; *χ*^2^ = 2.64, *p* = 1.26 × 10^−2^) and it occurred at a lower rate (Fig. 2c, Table 3, Dunn’s post-hoc test with Bonferroni correction; *χ*^2^ = −2.72, *p* = 9.68 × 10^−3^) when compared to active rejections. Indeed, interactions that resulted in active rejection were overwhelmingly terminated before the male secured a hold on the female’s tarsi (94.3%, Table 3). Our data suggest that active female rejection behaviours only significantly impacted outcomes up until this point, and 77.4% of pairs that ventrally aligned went on to complete copulation.

### Paired flight influences outcomes prior to ventral alignment

**Figure 3:**
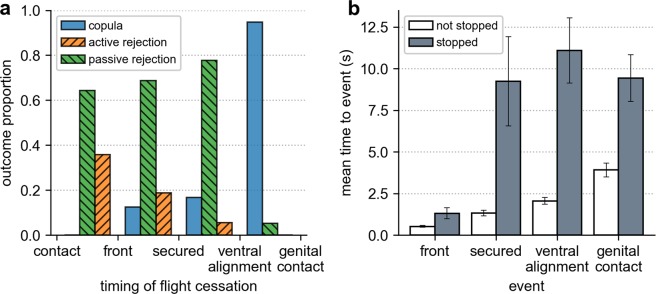
The effect of flight cessation on interaction outcome and progression speed. (**a**) The relative proportion of outcomes realised for interactions in which flight cessation took place between the labelled pre-copulatory behavioural events. Stoppage of flight overwhelmingly results in passive rejection, unless it occurs after the pair is ventrally aligned. Each interaction is only counted once. (**b**) The mean time from contact to event completion in all interactions where flight cessation did or did not occur at some point prior to the event. Vertical lines show the standard error. Note that “genital contact” data only considers those interactions that resulted in successful copula formation.

Flight cessation of one or both parties in the interaction occurred in 43.6% of observed interactions at a median time of 1.84 s post-contact (IQR 1.11–3.33 s), after the most intense phase of kicking activity (Fig. 2a). Females were more likely to cease flight before males, particularly in interactions that resulted in rejection (77.3%), in contrast to successful mating attempts (52.6%). The timing of flight cessation had a substantial effect on interaction outcome: if either individual stopped flying prior to ventral alignment, the attempt to form a copula was highly likely to passively fail (Fig. 3a, Table 1). This typically took place after the male had secured the female’s tarsi, but before the venter-to-venter position was adopted (64.9% of instances). Males would hang from the tethered female before flying away after a period of seconds. Once ventrally aligned, however, paired flight was not associated with mating outcome (Fig. 3a). Couples would readily engage in copulation with either one or both individuals stationary (61.0% of mating pairs), with the male holding the tethered female. Post-contact flight cessation at any point decreased the speed at which pairs progressed through each step of the interaction (Fig. 3b).

### Harmonic convergence

**Figure 4:**
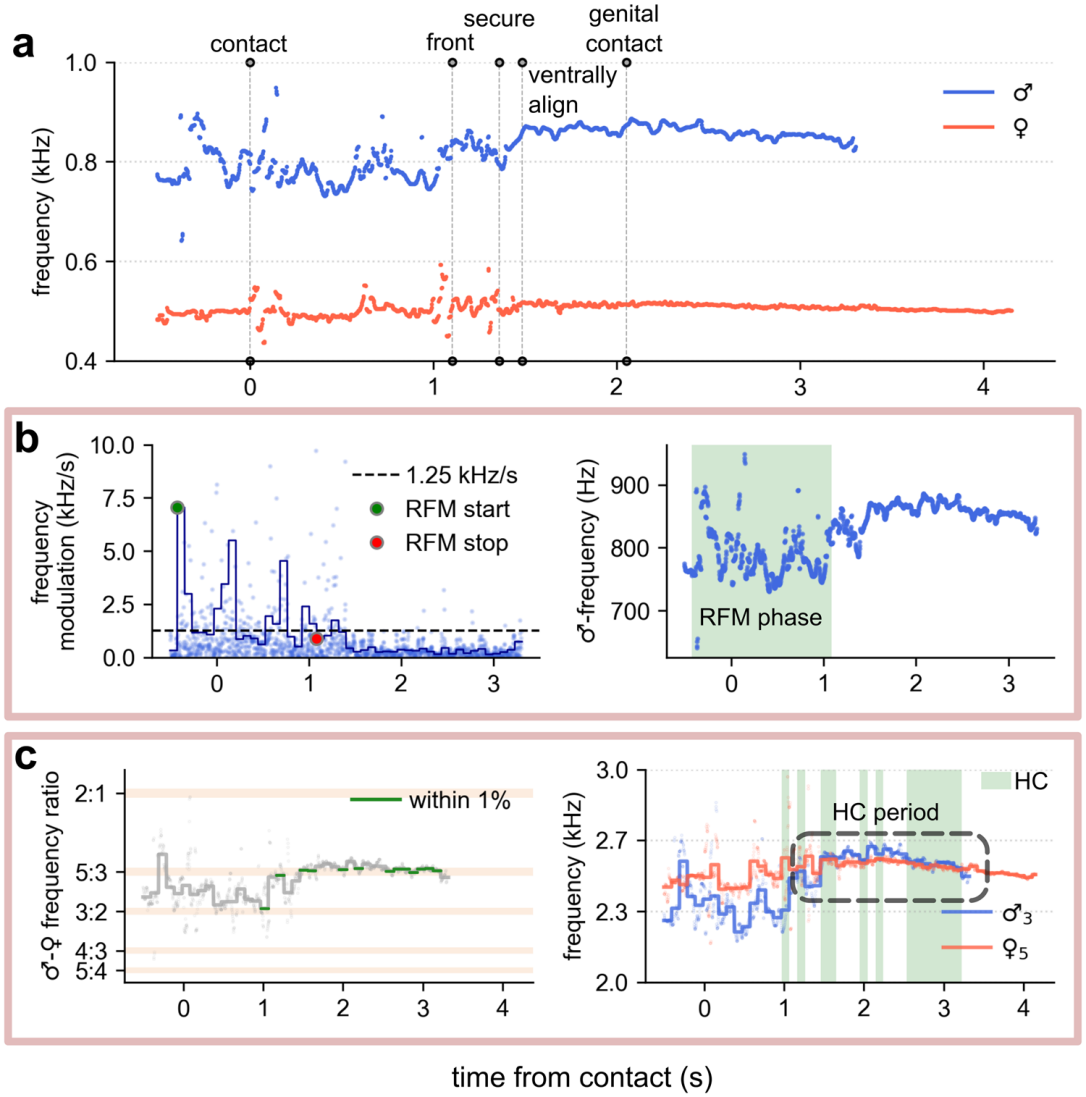
Wingbeat frequency measures of acoustic interactions for a mating attempt (in this instance one that resulted in copulation). (**a**) Male and female fundamental wingbeat frequencies extracted using the reassigned spectrogram method. Vertical dashed lines denote behavioural features of the interaction. (**b**) Detection of RFM^48,49^ in male flight tones. Left panel: point-to-point fundamental frequency modulation, overlaid with a smoothed average (blue line) equivalent to 0.08 s per segment. The dashed line shows the RFM threshold of 1250 Hz/s, with initiation and termination of the RFM phase given respectively by the green and red markers. Right panel: the male fundamental wingbeat frequency with identified RFM phase highlighted. (**c**) Automated detection of harmonic convergence events. Left panel: smoothed male-female fundamental wingbeat frequency ratio spectra with convergent segments—characterised as being less than 1% deviation from a given harmonic combination—highlighted in green. Right panel: male and female harmonic frequencies with the automatically quantified convergent periods highlighted in green. In this case, convergence was identified at the male 3^rd^, female 5^th^ harmonic overtone. Most harmonic convergence takes place when the wingbeat frequencies of both individuals are stabilised.

**Figure 5:**
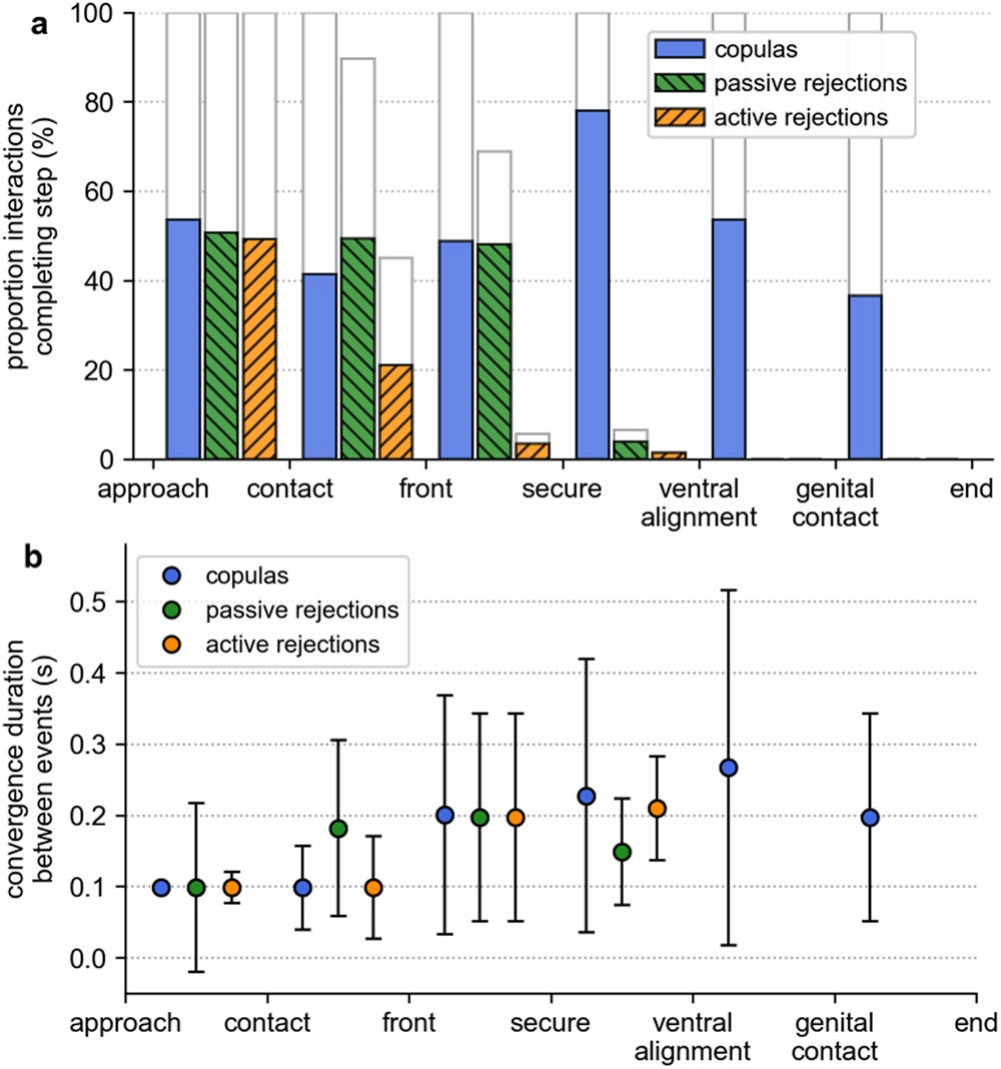
Harmonic convergence prevalence and duration at different pre-copulatory behavioural stages of interactions for different outcome types. (**a**) The proportion of all attempts completing each step. Coloured bars indicate the total proportion in which harmonic convergence was detected. (**b**) Median (±median absolute deviation) harmonic convergence durations between each behavioural step for all interactions, including those that did not complete the given stage. For interactions that ended during the period, convergence times were calculated over the interval beginning at the first event up to the point of termination.

Overall, harmonic convergence events (Fig. 4c) were less likely to be detected during interactions that terminated in active rejection than those that resulted in successful mating or passive rejection (Fig. 5, pairwise Fisher’s exact tests; *p* > 0.05). The total harmonic convergence duration was significantly greater in copulas (median 0.59 s, IQR 0.39–1.18 s) than in either active rejections (median 0.20 s, IQR 0.10–0.30 s, Dunn’s post-hoc test with Bonferroni correction; *χ*^2^ = 6.94, *p* = 5.94 × 10^−12^) or passive rejections (median 0.39 s, IQR 0.20–0.69 s, Dunn’s post-hoc test with Bonferroni correction; *χ*^2^ = 2.57, *p* = 1.54 × 10^−2^).

Prior to the ventral alignment stage, the incidence of harmonic convergence did not influence the likelihood of progression through the mating sequence (Fig. 5a, Fisher’s exact tests; *p* > 0.05). Copulas, however, displayed a significant peak in the incidence of convergence between the male securing the female’s tarsi and the pair becoming ventrally aligned (Pearson’s *χ*^2^ test; standardized residual of 3.65^56^), which was not present in unsuccessful attempts (Fig. 5a). The duration of convergence between successive pre-copulatory events was comparable across interaction outcome types (Fig. 5b), and was found to be positively correlated with paired flight time (Fig. S3). These data suggest that it is the timing, rather than the overall duration, of harmonic convergence that is associated with the increased rate of ventral alignment observed in successful mating pairs.

Rapid frequency modulation (RFM)^48,49^ of male flight tones was detected (Fig. 4b) in 260 of 265 (98.1%) contact-making interactions, beginning a mean (±standard deviation) time of 0.36 ± 0.24 s before interception of the female. The duration of RFM was strongly positively correlated with the total time that the pair spent in paired flight (Fig. S4, Spearman rank correlation; ρ = 0.91, *p* = 4.95 × 10^−102^). Thus, for mating attempts that resulted in rejection, the majority of the acoustic interaction (88.9%) was classified as RFM flight. In contrast, the conclusion of the RFM phase in copula-forming interactions coincided temporally with the adoption of the venter-to-venter position. In 73.2% of successful mating attempts, termination of RFM took place before ventral alignment was achieved (and after securing the female’s tarsi in 83.3% of these interactions).

Harmonic convergence events occurred both during and after the RFM phase in all outcome types. However, the relative proportion of total convergence time that was detected within RFM flight was significantly affected by the interaction result (one-way Analysis of Variance; *F*(2, 219) = 21.46, *p* = 3.09 × 10^−9^). Successful mating pairs exhibited a greater level of non-RFM convergence (40.5%) than active (9.1%) or passive (13.1%) rejection types (Tukey’s HSD post-hoc tests, *p* < 0.05). Sustained post-RFM flight (greater than 0.5 s) was more likely to be observed in attempts that ended in copulation (Fig. S4) than in either active (Pearson’s *χ*^2^ test; *χ*^2^ = 40.70, *p* = 5.33 × 10^−10^) or passive (Pearson’s *χ*^2^ test; *χ*^2^ = 9.01, *p* = 8.06 × 10^−3^) rejections. This period correlates behaviourally with ventral alignment and acoustically with a peak in harmonic convergence activity.

## Discussion

Copula formation in *Ae. aegypti* is a complex, dynamic process involving the integration of multiple behaviours mediated by rapidly fluctuating acoustic interactions. By analysing individual mating attempts with high precision, we were able to study the stages preceding copulation while simultaneously mapping acoustic features onto these behaviours at an unprecedented scale (Fig. 1). Our results demonstrate that female rejection responses strongly affect interaction outcome up until the critical point of ventral alignment. We observed that the maintenance of paired flight significantly affected adoption of the venter-to-venter position, and uncovered a high incidence of harmonic convergence localized around this transition in successful attempts.

Females were observed to exert a high degree of influence over the eventual outcome of a given mating attempt. As in other mosquito species^22,41,57,58^, rejection kicking was ubiquitously observed among mating attempts in *Ae. aegypti* (95.5% of all interactions contained female kicks). Displacement of the male through kicking resulted in his active rejection by the tethered female, which was the most commonly recorded interaction outcome (50.4% of attempts). While the majority of attempts involved some degree of kicking, females responded differently in attempts that eventually resulted in successful mating (Table 3, Fig. 2b,c). This variation in vigour strongly suggests that kicking forms the primary basis for post-contact female choice in *Ae. aegypti* courtship^59^. Physical female resistance to copulation is a common strategy in systems in which her choice plays a significant role in determining eventual mating outcome, and has been observed in a range of insect species^60–64^, as well as in birds^65,66^ and reptiles^67,68^. It is thought that these behaviours may enable the female to assess male quality prior to insemination^61^.

The majority of kicks are delivered within the first few seconds of male contact (Table 3, Fig. 2a). Males that endured this initial phase and manoeuvred into a secured position ventral to the female increased their chances of mating with her: 77.4% of pairs that ventrally aligned copulated (Table 1). Most pairs assumed the venter-to-venter position in flight (84.9%), and while—as in free-flying mating observations^18,21^—genital contact and engagement occurred with one or both individuals stationary, it was always more likely and occurred quicker when paired flight was sustained (Fig. 5b). Flight cessation before ventral alignment commonly resulted in termination of the attempt (Fig. 5a). Previous observations of free-flying mating interactions of *Ae. aegypti* reported high rates of failure among pairs that landed before adoption of the venter-to-venter position^21^. In natural swarms, couples are readily seen to quickly exit the group once in contact with one another^11^. While this could be to prevent interference from competitors, it is also possible that females seized by an unwanted male could steer towards the ground or nearest substrate to terminate the interaction. If this were the case then we would anticipate the paired-flight interval in these couples to be shorter than those resulting in successful copulation, which is indeed what our results indicate (Table 3). In our observations, mating attempts that failed due to cessation of paired flight were recorded as passive rejections (27.3% of interactions). As this was a primarily female-initiated behaviour, we suggest that it offers a plausible means through which females can deter males that are not displaced by rejection kicks.

The rates of copula formation we detected are comparable to previous studies of tethered *Ae. aegypti*^40^. Our findings suggest that there is only a relatively brief window of opportunity over which rejection behaviours are effective. Male mosquitoes have a high capacity to repeatedly attempt copulation^18^, and the majority of mating pairs we observed were formed after the first contact-making attempt. Male harassment has little effect on the lifetime fitness of a given female^69^, but it is possible that—within a given mating swarm—females become more receptive the more times she is intercepted. Our data offer some evidence for this effect: the number of kicks delivered by a female decreased, on average, with increasing contacts (Table S1). In a typical swarm environment, where numerous males compete for a relatively small number of females, males may optimise their participation based on their own energy reserves, mating ability, and the peak of female receptiveness^36^.

Our results also refine our understanding of the role of sound in mosquito mating interactions. Males approached and contacted tethered females predominantly from frontal and dorsal directions (Table 2), corroborating recent observational data from free-flying mating couples^41^. The flight sounds of female *Ae. aegypti* are known to be loudest ahead and behind her^70^; behaviourally, our observations show that this coordinates the final movements of the male prior to interception of the female. We explored two aspects of the acoustic interaction between pairs: rapid frequency modulation (RFM)^48,49^—described for the first time here in *Ae. aegypti*—and harmonic convergence^52^.

RFM was highly stereotyped across almost all interactions, coinciding with the timing of the initial grab by the male and lasting through to the point of ventral alignment (or the termination of paired flight, whichever came first). Temporally, the RFM phase spans the most turbulent period of the interaction, encompassing the bulk of the female-kicking and male-manoeuvring phases. The onset of RFM may be a consequence of the male attempting to maintain a controlled flight position in close proximity to the female to facilitate her seizure^49,71^. However, the fact that it continues well into the pre-copulatory behavioural sequence suggests it also manifests due to physical processes disruptive to the maintenance of stable flight. This assertion is supported by the observation that female wingbeat frequencies displayed a corresponding RFM-like phase in the post-contact period (Fig. 4a), with the flight tones of both individuals stabilising at the offset of RFM and the adoption of the venter-to-venter position.

Our findings support the notion that harmonic convergence is a distinct process to RFM^49^. Its occurrence was not constrained to a particular stage of the pre-copulatory behavioural sequence, and there was little to differentiate copulas and rejections in terms of their convergence activity in the early phases of an interaction (Fig. 5). The interaction between convergence and other behavioural features was non-trivial (Table S2). In contrast to unsuccessful mating attempts, pairs that completed copulation displayed a peak in harmonic convergence activity over the interval between the male securing the female’s tarsi and the pair becoming ventrally aligned (Fig. 5a). This coincides with the end of the kicking period and the termination of RFM. The lack of an effect of convergence on female kicking behaviour up to this point and the high rate of copulation observed post-ventral alignment support the hypothesis that, rather than forming the basis of an acceptance decision, convergence is a manifestation of physical co-ordination in the final moments preceding copula formation (i.e. adoption of the venter-to-venter position)^43^. Convergence may enable or result from adjustments in position as the pair seek to stabilise and control their relative orientations mid-air. It is worth noting that the mechanisms of intraspecific post-contact copula formation that may involve harmonic convergence are likely distinct from those proposed for interspecific mating isolation^54^.

Alternatively, the timing of convergence could play a role in facilitating ventral alignment. In many mating systems, females assess the fitness of males through a complex interactive encounter. The ability of a male to provide an appropriate signal at a precise moment in time can reveal information pertaining to his physiological state^59^. In the acoustic domain, this would require an acute sense of hearing. Current models of mosquito audition suggest that male responses to sound are driven by differences between the frequencies generated by their own wings and those of a nearby female^46,53,71^, enabling him to “lock into” and amplify her flight tone^72^. The fine-scale modulation of frequencies required to achieve harmonic convergence at just the right moment to enact ventral alignment would then be facilitated by this highly tuned system.

In mating aggregations such as mosquito swarms, males are often evaluated based on behavioural, rather than morphological, traits^73^. Variation within such systems is high, with fitness indicators often demanding the integration of several physiological processes. Our results reveal an intricate and diverse pathway towards copulation in mosquitoes. The tethered arrangement employed in this study may have restricted the range of behaviours that would normally be displayed in a free-flying mating attempt. For example, females were unable to perform evasive flight manoeuvres, and their sensory experience throughout the interaction may have been altered because of their positional fixation. However, this assay offers a means to investigate this process with high precision. The findings we present serve as an important benchmark towards developing an understanding of how behavioural features of mating attempts interact with other phenotypic characteristics, such as body size^43^, and their genetic basis^40^, in free-flying pairs. In addition, our work establishes fresh perspectives on the chronology and function of harmonic convergence in copulation attempts. This information will ultimately provide insights into mosquito mating biology that can be used to increase the effectiveness of mass-release lines.

The tools developed in this study open the possibility to study both the physical and acoustic features of mosquito mating interactions at a level of detail that has not before been possible. Our results implicate female choice as a decisive factor in determining male mating success of *Ae. aegypti*. Females reject males by delivering tarsal kicks to displace them, or through the cessation of flight to terminate movement co-ordination. The majority of mating attempts are unsuccessful. Males that mated were kicked at a lower frequency, and remained in paired flight for longer, than those that were rejected. Our observations highlight adoption of the venter-to-venter position as the critical barrier towards achieving genital contact. This orientation was, in most instances, achieved while both individuals were beating their wings. In this regard, our findings suggest a more subtle role for harmonic convergence than has been indicated in previous studies^49,50,52,53^, with a peak in convergence activity occurring well after initial contact and coinciding with the critical move to ventral alignment in mating pairs. While further work is needed to clarify the relative function of these processes in individual free-flight interactions, how they are affected by the pressures of competition, and whether they can be selected for in laboratory lines, our findings offer a platform from which to target specific aspects of mosquito reproductive behaviours in future investigations.

## Methods

### Mosquito husbandry

We hatched *Ae. aegypti* eggs (F2-3, originating from Kampheang Phet, Thailand) by submerging them in water held under vacuum for 20 minutes. Hatch flasks were given a small amount of larval food (Cichlid Gold, Hikari, Kyrin Food Industries Ltd, Japan) and left inside a climate-regulated incubator (27 °C, 80% relative humidity) for approximately 18 hours. First-instar larvae were then sorted into groups of 250 and placed into containers filled with 500 ml distilled water. Larvae were fed a diet of 0.5 mg/larva/day throughout development. Pupae were transferred individually into 15 ml falcon tubes plugged at the top with cotton wool and monitored daily for the emergence of adults. Each evening, males and females were released into sex-segregated and day-specific acrylic flight cages (20 cm^3^). Adults were offered a 10% sucrose solution and held at 27 °C, 80% relative humidity on a D12:N12 (with 30 minutes of dawn/dusk separating each phase) circadian cycle.

### Semi-tethered recording assays

We simultaneously captured behavioural and acoustic data of interactions between males and females using a custom-built recording arena. Individual 3–7 day old virgin females were extracted from flight cages and were, under cold narcosis (induced by anaesthetising on ice for 30 s), glued (Nailene, Pacific World Inc., Aliso Viejo, CA, USA) to a short (~20 mm) strand of human hair (LJC) affixed to a metal pin. This pin was mounted on a stand in the centre of the recording chamber, a 20 × 20 × 20 cm white-mesh enclosure with an acrylic front face to enable video recordings (Fig. S1). The cage was set on top of a heating plate to increase the local temperature throughout experiments (25 ± 1.8 °C, 28.1 ± 2.0% RH monitored using an EasyLog USB data logger, Lascar Electronics Ltd, Whiteparish, Wiltshire, UK). After allowing the female sufficient recovery time (1–3 minutes), we stimulated flight by gentle blowing or through tarsal contact. Stability of female flight was confirmed by adoption of the characteristic airborne position (Fig. S1). Once in this position, tethered females will fly consistently for over 4 hours before reaching exhaustion^74^. In this study, females were exposed to males within 15 minutes of recovery. In natural populations of *Ae. aegypti* mating takes place around the human blood-meal host^10,11^. Host stimulation was therefore provided by placing a worn t-shirt (AA) between the recording arena and the heating plate, and by standing close to the flight enclosure (<30 cm) during experiments. The trial was initiated by releasing a single 3–7 day old virgin male into the cage through an access hole on the front face (Fig. S1). Males that settled on the sides of the arena were encouraged to fly by physical disturbance. All experiments were conducted under ambient lighting conditions between 08:00 h and 17:00 h.

The flight tones of each insect were recorded using a pair of microphones; one, a directional particle velocity microphone (NR-23158, Knowles Electronics, Itasca, IL, USA), was placed directly underneath a custom-made pop filter (to prevent acoustic clipping interference caused by male contact with the microphone) positioned below the tethered female at a distance of roughly 2 cm. The other, an omnidirectional electret condenser microphone (FG-23329, Knowles Electronics, Itasca, IL, USA), was mounted approximately 4 cm behind the female (Fig. S1). Data from this second microphone was used in analyses where recording quality on the primary microphone was poor. Microphone signals were passed through a integrating multi-channel sound amplifier^75^ and logged digitally using a data acquisition device (NI USB-6212, National Instruments, Austin, TX, USA) at a sample rate of 10 kHz. A high-speed digital camera (Phantom Miro 310, Vision Research, Wayne, NJ, USA) was used to film proximate interactions (frame size on the order of 10 mosquito body-lengths) between the pair at a rate of 1000 fps. Experiments were carried out in natural lighting conditions, with the arena illuminated using a single red-light multi-LED lamp (GS-Vitec 7700 lm, Vision Research, Wayne, NJ, USA) positioned around 2 m in-front of the recording booth. Audio and video data streams were synchronously recorded using Phantom Camera Control (PCC, Vision Research, Wayne, NJ, USA) software.

A configurable software trigger was set to initiate recordings at the first attempt of the male to contact the tethered female. The preceding 12 s and subsequent 40 s of interaction were recorded around this point, yielding a total of 52 s of recorded data—video and audio—per pair, over a distance of approximately 10 body lengths (around 5–7 cm). This experiment was repeated with two replicates containing, respectively, 52 and 65 unique pairs (117 total). All trials resulted in some form of contact attempt. Females from pairs (in the second replicate) adjudged to have copulated successfully were dissected to assess insemination rates. All females were found to have been successfully mated. Note that individual mosquitoes were used only once for a single recording and were not re-used across trials.

### Data processing: video and audio streams

High-speed video recordings of short-range (<5 cm) mosquito interactions were analysed manually to determine various qualitative and quantitative features (Table 1), which were precisely time-stamped by frame number and, where relevant, counted.

Extraction of mosquito flight tones involved a multi-step fundamental-frequency estimation process^76^. First, we computed the short-time Fourier Transform (STFT) of a given recording, using a spectral reassignment correction^77,78^ to enhance resolution in the frequency and time domains^79^. To isolate individual male and female wingbeat frequencies, an *a priori* estimate of each mosquito’s fundamental flight tone bandwidth was subsequently used to constrain the resulting frequency spectrum. A point-by-point reconstruction of the primary frequency component within each channel was then achieved by determining the frequency band corresponding to the maximum average power spectral density of a sliding window containing 10 evenly-spaced time frames (with the window overlap set to 90%). The use of a sliding window to determine instantaneous frequencies increased the resilience of the approximation to local spectral instabilities^76^. The output from this procedure was a set of 1-dimensional time series representing the male and female fundamental flight tones over the course of a single recording. A final step involved visual inspection of the extracted signals to confirm their integrity, and manual pruning to remove bouts of non-flight (Fig. 4a).

We extracted male and female wingbeat frequency data associated with a given interaction to cover the period up to 1 s before contact, extending up to the point of physical separation, ensuring an audio separation between successive interactions of at least 0.1 s. We then characterised two measures of acoustic behaviours intended to capture different features of this signalling domain.

#### Rapid frequency modulation (RFM)

As a male mosquito approaches a female-like sound source, his wingbeat frequency is observed to rapidly modulate for a sustained period of time^48,49^. We measured the duration and start–stop points of this acoustic behaviour in recorded males. Rapid frequency modulation involves modulations in flight tone of greater than 1250 Hz/s occurring at a rate of 12.5 modulations per second^49^. With these criteria, we calculated the average frequency modulation of a given male using windowed increments equivalent in length to 0.08 s. Where this average exceeded 1250 Hz/s, RFM was deemed to have begun (Fig. 4b). The termination of RFM was identified by a period (0.5 s) of flight whereby frequencies deviated by less than the RFM threshold (Fig. 4b).

#### Harmonic convergence

We assessed harmonic convergence activity using an “automated” method to quantify the number and precise timing of individual harmonic convergence events. The male-to-female fundamental wingbeat frequency ratio was smoothed using a windowed average^80^ 0.1 s in length. Unique instances of harmonic convergence were then identified as segments that deviated by less than 1% of integer overtone combinations previously reported to denote frequency interactions^50^ (Fig. 4c).

### Data Analyses

The video and acoustic data streams were combined and aggregated over all recordings to produce a generalised ethogram of the *Ae. aegypti* mating sequence (Fig. 1). We then compared observational and behavioural features (approach and contact direction, interaction duration, total number of kicks, time from contact to first kick, duration of kick period, kick rate, time from contact to male frontal positioning, post-contact paired flight period) of mating attempts that ended in the main outcome types (successful copulation, active rejection, and passive rejection, Table 1) to investigate quantitative differences between interactions. Variables were checked for normality using the Shapiro-Wilk test; subsequently, non-parametric methods were used to compare distributions^81^. Specifically, we applied the Kruskal-Wallis test to analyse differences in particular variables across the interaction outcome types. Where appropriate, Dunn’s tests were then used to make pairwise post-hoc comparisons. Fisher’s exact test was used to investigate the relationship between approach and contact direction. To explore the relationship between female kicking and interaction outcome, we used the *χ*^2^ test for independence with Monte-Carlo resampling (due to low count frequencies). To compare the incidence of harmonic convergence across outcome types at each behavioural mating step we used Fisher’s exact test (number of converging versus non-converging pairs), adjusting *p*-values with the Bonferroni correction due to multiple comparisons (across steps). Analysis of standardised residuals in the Pearson’s *χ*^2^ test enabled identification of significant peaks in harmonic convergence incidence of copula forming interactions. We used a threshold magnitude of ≥3^56^ to infer significance. The proportions of harmonic convergence occurring within and outside the RFM phase were compared across outcome types using one-way Analysis of Variance, with Tukey post-hoc tests implemented to explore pairwise differences. Finally, pairwise Pearson’s *χ*^2^ tests were used to explore differences in sustained post-RFM flight between the main interaction outcome types (number of interactions with at least 0.5 s of post-RFM paired flight); *p*-values were adjusted using the Bonferroni correction to account for multiple comparisons. All statistical tests were performed in R^82^.

## Supplementary information

Supplementary information

## Data Availability

The datasets collected and analysed during this study are available from the corresponding author on reasonable request.
