# Supplementary material for: Female resistance and harmonic convergence influence male mating success in Aedes aegypti
Source: Sci Rep. 2019 Feb 14;9:2145. doi: 10.1038/s41598-019-38599-3 (PMC6375921; doi:10.1038/s41598-019-38599-3)
Supplement: Supplementary file 1 — Supplementary information [file 41598_2019_38599_MOESM1_ESM.docx]

**Supplementary Information for “Female resistance and harmonic convergence influence male mating success in *Aedes aegypti*”**

Andrew Aldersley^1^, Lauren J. Cator^1^

^1^ Department of Life Sciences, Imperial College London, Silwood Park, Buckhurst Road, Ascot, SL5 7PY, UK

| Contact attempt | Number of pairs | Mean (± sd) number of kicks |
| --- | --- | --- |
| 1^st^ | 113 | $6.41\pm4.99$ |
| 2^nd^ | 71 | $5.35\pm4.07$ |
| 3^rd^ | 40 | $4.65\pm3.75$ |
| 4^th^ | 16 | $2.75\pm1.48$ |

**Supplementary Table S1** Mean (± standard deviation) number of kicks delivered by females by contact number. For a given pair, males receive fewer kicks with each successive contact attempt with the female.

| Event A | Event B | Outcome | | | | | |
| --- | --- | --- | --- | --- | --- | --- | --- |
|  |  | Copula | | Passive rejection | | Active Rejection | |
|  |  | C | NC | C | NC | C | NC |
| Contact | Move front | 0.39 | 0.23 | 0.62 | 0.18 | 0.44 | 0.23 |
| Move front | Secure | 1.11 | 0.18 | 0.91 | 0.16 | 0.98 | 0.15 |
| Secure | Ventral alignment | 1.44 | 0.45 | 1.52 | 0.29 | 1.09 | - |
| Ventral alignment | Genital contact | 1.83 | 0.96 | - | - | - | - |

***Supplementary Table S2*** Median time taken (in seconds) to complete the transition from “Event A” to “Event B” for different interaction outcomes in which harmonic convergence was identified (C), versus those in which harmonic convergence was not identified (NC).


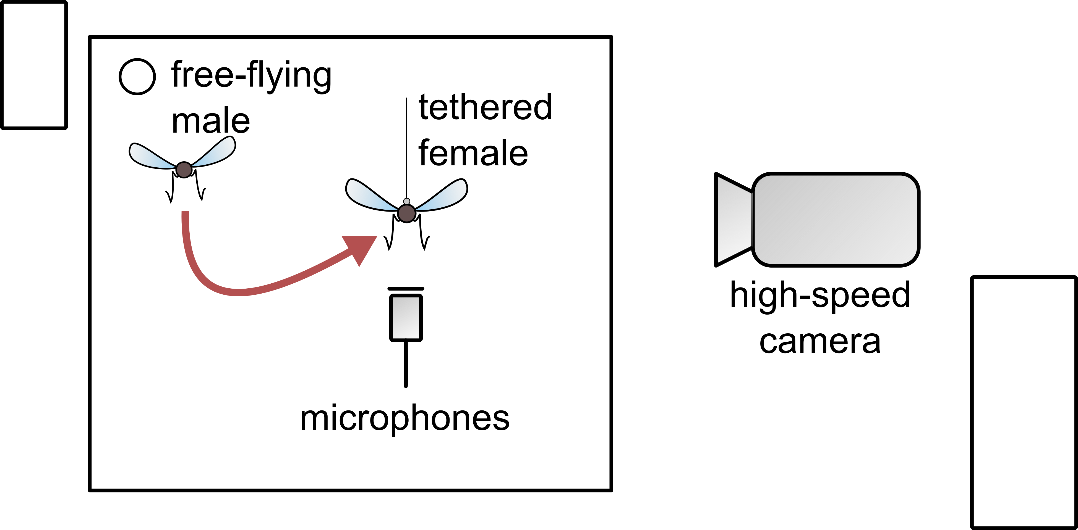


**Supplementary Figure S1** Diagram showing the layout of the recording arena used for semi-tethered mating experiments. A single female Aedes aegypti mosquito is tethered to a strand of human hair and positioned above a microphone, which is synchronised with a high-speed video camera. A lone free-flying male is then released into the arena. Male-female interactions are captured simultaneously on video and audio channels.


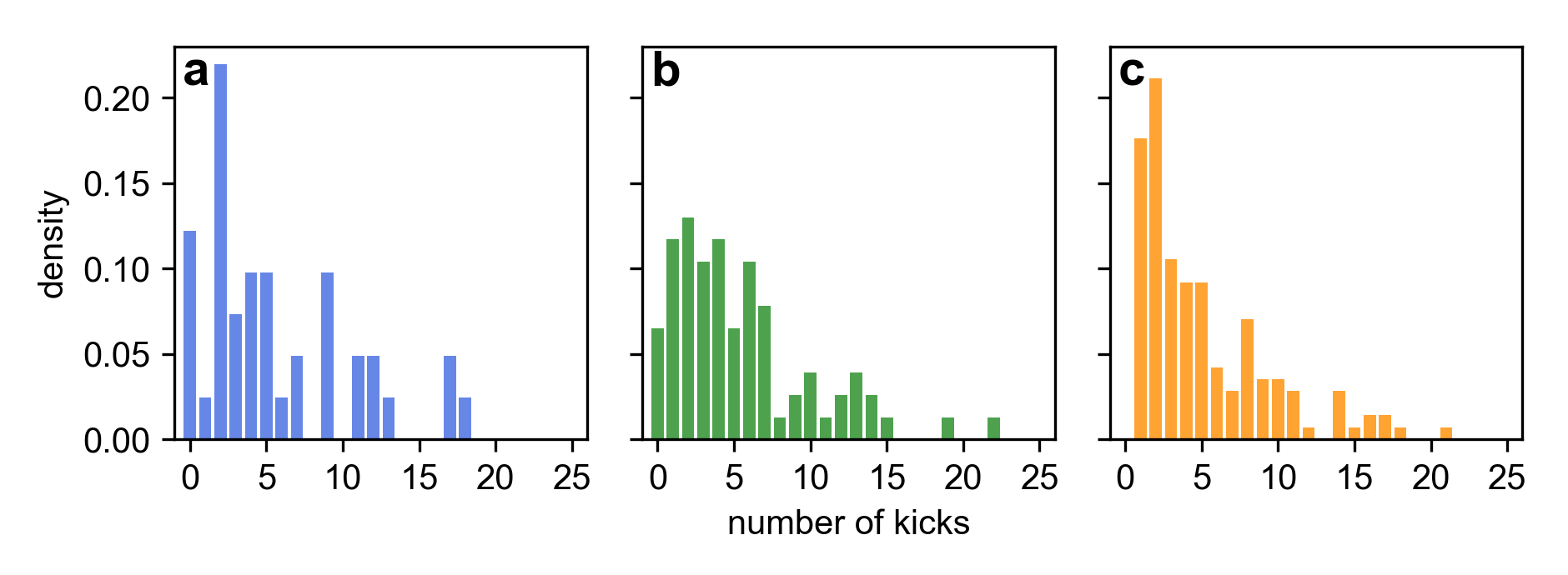


**Supplementary Figure S2** Distribution of number of kicks for (a) copulas, (b) passive rejections, and (c) active rejections.


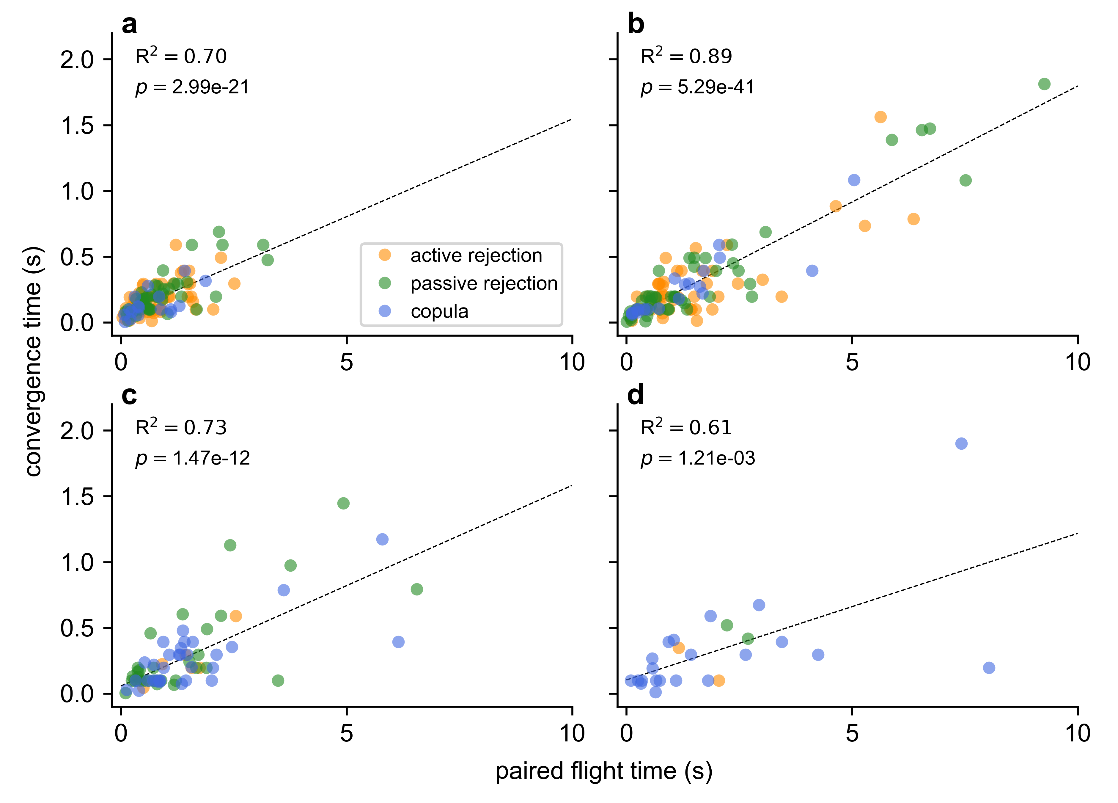


**Supplementary Figure S3** Harmonic convergence duration plotted as a function of paired flight time for the intervals between pre-copulatory behavioural events (a) contact and the male moving to the female’s front, (b) moving to the female’s front and securing her tarsae, (c) tarsal securing and ventral alignment, and (d) ventral alignment and genital contact. Note that in interactions where the first event was completed but the second was not (i.e. in active and passive rejections), paired flight time and harmonic convergence durations were calculated for the period from the first event to the termination of the interaction. Lines of best fit were calculated using linear least-squares regression. Also shown is the correlation coefficient (R^2^) and associated p-value.


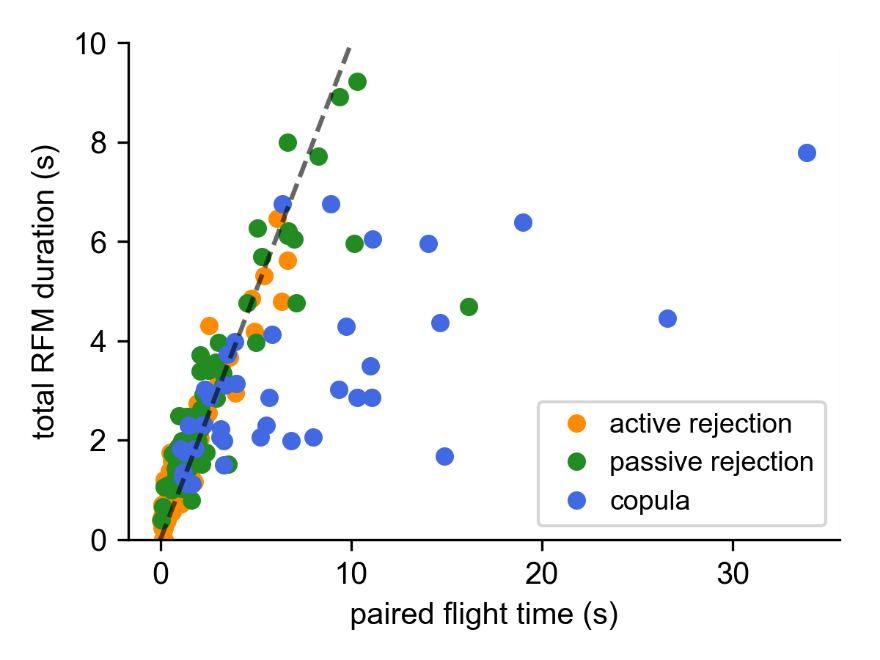


**Supplementary Figure S4** The total duration of rapid frequency modulation (RFM) plotted as a function of paired flight time (while individuals are in contact with one another) for all interactions. Dashed line is representative of a 1:1 relationship. For interactions with a shorter paired flight time (the majority of which are active and passive rejections) the RFM phase essentially constitutes the entire acoustic signal. When the pair remain in flight for longer, as is often the case in interactions that result in copula formation, the RFM period is proportionately shorter, and is followed by a prolonged phase of smooth, stable flight with characteristic low-modulation wing beat frequencies (Figure 4bc).

***Supplementary Videos*** are available at https://imperialcollegelondon.box.com/v/aldersley-cator-2018

***Supplementary Video S1*** Male approach, hover, and tarsal grab to intercept tethered female (video slowed to 5% actual speed).

***Supplementary Video S2*** After contact, the pair entered a phase of manoeuvring during which the male would crawl to the front of the female, secure her tarsi with his own, and attempt to ventrally align (Table1, Figure 1) with the female (video slowed to 10% actual speed).

***Supplementary Video S3*** Females delivered tarsal kicks and thrusts to any male that made contact with them, which would often cause the male to be displaced. Successful removal of the male was referred to as active rejection (Table 1, video slowed to 10% actual speed).

***Supplementary Video S4*** Copulas were recorded whenever genital contact was made, regardless of whether insemination took place (Table 1). This example begins with the pair already ventrally aligned whilst in paired flight (video slowed to 10% actual speed).

***Supplementary Video S5*** Interactions that were classified as passive rejection were terminated by flight cessation (Table 1), typically instigated by the female (video slowed to 10% actual speed).
